# Retrospective, Observational, Pilot Study to Assess the Safety and Efficacy of Goel’s Technique of Laparoscopic Hysterectomy in Endometrial Carcinoma

**DOI:** 10.7759/cureus.71525

**Published:** 2024-10-15

**Authors:** Vipin Goel, Bushra Khan, Snigdha Rampelly

**Affiliations:** 1 Department of Surgical Oncology, Star Hospitals, Hyderabad, IND

**Keywords:** endometrial carcinoma, goel's technique, hospital stay, laparoscopic hysterectomy, postoperative complications

## Abstract

Background

Laparoscopic hysterectomy is a substitute for the abdominal hysterectomy technique for endometrial carcinoma. Goel’s technique is a unique laparoscopic hysterectomy. The main feature of Goel’s technique is that vaginal manipulators or myoma screws are not used in the procedure as vaginal manipulators or myoma screws contribute to an increased risk of spread of malignancy in the systemic circulation.

Methods

In this retrospective, observational, pilot, single-centre study, the patient’s baseline demographics, clinical characteristics, and assessment and outcome measures of Goel’s technique of laparoscopic hysterectomy were recorded. The following metrics were used to assess the postoperative recovery: average time to discharge the patients after the surgery; postoperative complications/pain assessment; correlation between pain and day of hospital discharge; association between the day of discharge and postoperative pain assessment; association between operation time and complications.

Results

A total of 35 female patients with early-stage endometrial cancer were included, their mean age being 56.29 years. The mean time to hospital discharge of the patients was 2.94 days. Of the cases, 2.9% had a ureteral injury and ureterovaginal fistula as complications, which were resolved during the follow-up period. On day one, the mean pain score decreased significantly to 50% from day zero (D0), and on day two, the mean pain score showed a significant fall of 91.5% from D0. Ten patients with a mean pain assessment score of 3.60 at D0 were released on the second day, 20 patients with a mean score of 3.80 at D0 were released on the second day, and five patients with a mean score of 5.60 at D0 were released at ≥ four days. Not a single patient developed any wound infection, dehiscence, or herniation for six months.

Conclusion

Goel’s technique helped patients with endometrial carcinoma to recover faster and it reduced hospital stays with fewer postoperative complications.

## Introduction

Endometrial cancer is the sixth most commonly occurring cancer in women with an incidence rate (world) of 13.3 per 100,000 women [1]. Though cervical cancer remains the leading gynaecological cancer in developing countries, recently there has been an increase in the incidence of endometrial cancer. In India, the age-standardized incidence rate (ASIR) of endometrial cancer is 2.3/100,000 women [2]. Total abdominal hysterectomy (TAH) and pelvic lymphadenectomy are standard lines of treatment for endometrial cancer as far as surgical staging is concerned. Lymphadenectomy, including the removal of the para-aortic lymph glands, is commonly used for non-endometrioid cancers, poorly differentiated tumours (Grade 3), invasion of >50% of the myometrium, or noticeable involvement of the pelvic lymph glands [3].

There are three options to carry out the hysterectomy: vaginal hysterectomy, abdominal hysterectomy, and laparoscopic hysterectomy [4-6]. Out of these, the extensively used surgical technique is abdominal hysterectomy. Abdominal hysterectomy is recommended for endometriosis, uterine adhesions, and enlarged uterus. As compared to abdominal hysterectomy, vaginal hysterectomy is a lesser invading technique. A vaginal hysterectomy is mostly recommended for uterine prolapse. A vaginal hysterectomy is also used for normal-sized uterus nowadays to treat cases with menstrual abnormalities [6]. The abdominal hysterectomy can be replaced with a laparoscopic hysterectomy. In laparoscopic hysterectomy, the following things are done: main vessels of the uterus are tied off, the correctness of dissection is ensured, ureters are identified, and careful haemostasis is achieved [6,7]. As compared to vaginal and abdominal hysterectomy, laparoscopically assisted vaginal hysterectomy needs more surgical expertise and because of this, laparoscopically assisted vaginal hysterectomy has a lesser reception in the gynaecology segment as compared to various other surgical disciplines. The following factors also contribute to lesser reception of laparoscopically assisted vaginal hysterectomy in the gynaecology segment: extensive operation time, lack of training, drought of enthusiasm to accept innovations among gynaecologists, and funding for the procedure [5,7-9]. This procedure has several benefits though the operation time is lengthier. This technique is preferred for treating endometriosis, performing adnexal surgeries, ovarian amputations, and securing problematic intraperitoneal bleeding cessation. Compared to abdominal hysterectomy, laparoscopically assisted vaginal hysterectomy offers less blood loss, fewer wound complications, a shorter hospital stay, and a quicker recovery for patients [5-11].

For the treatment of endometrial and cervical cancer, laparoscopic hysterectomies have been performed more often in recent years [7]. Though several different techniques for performing laparoscopic hysterectomies are used, most of these procedures are similar to each other [5,7,9,10]. There is a difference in the order of operative steps followed while doing various laparoscopic hysterectomies. These variations can have their advantages and disadvantages.

To manipulate the uterus for traction, vaginal manipulators are used during laparoscopic hysterectomy. However, it can lead to tissue breakup and bleeding and spread the malignancy if manipulators enter into cancer cells present in the endometrial cavity [6]. Goel’s technique is unique as it follows 10 fixed operative steps sequentially while performing a laparoscopic hysterectomy to ensure safety [12]. Before cutting down, the identification of anatomical structure is emphasized. The use of vaginal manipulators/myoma screws has the risk of spread of malignancy through the systemic circulation. Therefore, vaginal manipulators or myoma screws are not used in the Goel’s technique.

To confirm the usefulness of Goel’s technique while performing laparoscopic hysterectomy, we did a retrospective evaluation of the patients who underwent the procedure for the management of endometrial carcinoma. These patients were evaluated for the postoperative recovery journey.

## Materials and methods

Study objective

Verifying the effectiveness of Goel's approach (technique) during laparoscopic hysterectomy was the study's main goal.

Study design

This was a retrospective, observational, pilot study in a real-life clinical setting done at a single centre in patients suffering from endometrial carcinoma. The data were gathered for patients who had Goel's method (technique) laparoscopic hysterectomy between July 14, 2020, and December 21, 2022.

Evaluation parameters

Patients’ baseline demographics, clinical characteristics, and assessment and outcome measures were recorded at baseline and three to four days postoperatively. These patients were evaluated for the postoperative recovery journey by using the following parameters: average time to discharge the patients after the surgery; postoperative complications; postoperative pain assessment; correlation between pain and day of discharge; association between the day of discharge and postoperative pain assessment; wound healing; association between operation time and complications. Patients were followed up for six months for wound healing.

Statistics

Study parameters were evaluated three to four days after surgery by using the Wilcoxon sign rank test, Fisher's exact test, and Pearson correlation coefficient test for postoperative pain assessment, an association between operation time & complications, and correlation between pain & day of discharge, respectively. When the p-value was ≤0.05, it was deemed statistically significant. SPSS version 10.0 (SPSS Inc., Chicago, IL) was used for statistical analysis.

Ethics

This study was conducted following the standard procedures/protocols of conducting retrospective studies [12]. All ethical principles were followed while conducting this study.

Goel’s technique of laparoscopic hysterectomy and operative procedure

Dr. Vipin Goel first detailed and experimented with this laparoscopically assisted vaginal hysterectomy technique and therefore it is named after him. Goel’s technique is comprised of 10 steps. The first important and differentiating step starts with the placement of ports without vaginal manipulators or myoma screws. Dr. Goel has explained all the steps in another publication. Before performing a hysterectomy, Goel’s technique focuses more attention on the separation of the bladder, ureter, and rectum at a safe level. Goel’s technique helps to decrease the complication rate to a minimum [13].

Laparoscopic energy sources have been introduced for haemostasis and dissection to get over the difficult nature of laparoscopic suture ligation [14,15]. The Advanced Hemostasis (AH) device was developed in 2013 by Ethicon Endo-Surgery, Inc. (Cincinnati, OH). It utilizes the Harmonic ACE^TM^ Shears (Ethicon Endo-Surgery, Inc.) combined with a versatile adaptive tissue innovation (ATT) calculation to screen the instrument and react scholarly to tissue conditions. ATT gives more exact vitality conveyance, leading to less thermal impairment, fewer attachments (adhesions), speedier transection, less visual hindrance, and higher burst weights (pressures), as illustrated in preclinical trials [16]. This algorithm was further optimized by the AH device. For bigger vessels up to 7 mm in size, the advanced haemostasis mode (AHM) is intended. Haemostasis is increased and cutting speed is further decreased in this setting [17].

## Results

Demographic data

This study comprised 35 patients with endometrial cancer that was still in the early stages. As shown in Table 1, the mean age was 56.29 ± 08.43 years (range = 38-74 years). In this study group, 51.4% of study cases belonged to the age group of 51-60 years, 22.9% belonged to less than or equal to 50 years, 22.9% belonged to 61-70 years, and 5.7% of cases were more than 70 years old.

**Table 1:** Demographic data.

Age group (years)	No. of cases (N = 35)	Percentage (%)
≤50	08	22.9
51-60	18	51.4
61-70	07	20.0
>70	02	05.7
Average age	56.29 ± 08.43	

Average time to discharge the patients after the surgery (hospital stay)

The study results reveal that the mean time to discharge the patients after surgery was 2.94 days. Of the patients, 28.6% were discharged on day two, 57.1% on day three, and 14.3% of patients on day four or more, as demonstrated in Table 2.

**Table 2:** Average time to discharge the patients after the surgery (hospital stay).

Duration in days	No. of cases (N = 35)	Percentage (%)
2	10	28.6
3	20	57.1
≥4	05	14.3
Mean ± SD	02.94 ± 00.87	

Operative time

The mean operative time was 132.6 minutes (120.0-240.0 minutes).

Postoperative complications

This study indicates that 2.9% of the cases had a ureteral injury and ureterovaginal fistula as a complication, as shown in Table 3, and all these complications were resolved during the follow-up period. None of the patients had a blood transfusion, bowel injury, bladder injury, or vesicovaginal fistula. However, one patient had delayed recovery as she developed postoperative ileus. In addition, these patients were followed up for six months and it was noted that not a single patient developed any wound infection, dehiscence, or herniation.

**Table 3:** Postoperative complications.

Complications	No. of cases (N = 35)	Percentage (%)
Ureteral injury	01	02.9
Vesicovaginal fistula	00	00
Bowel injury	00	00
Blood transfusion	00	00
Bladder injury	00	00
Ureterovaginal fistula	01	02.9
Total no. of patients	01	02.9
Total no. of events	02	05.7

Changes in postoperative pain

**Figure 1:**
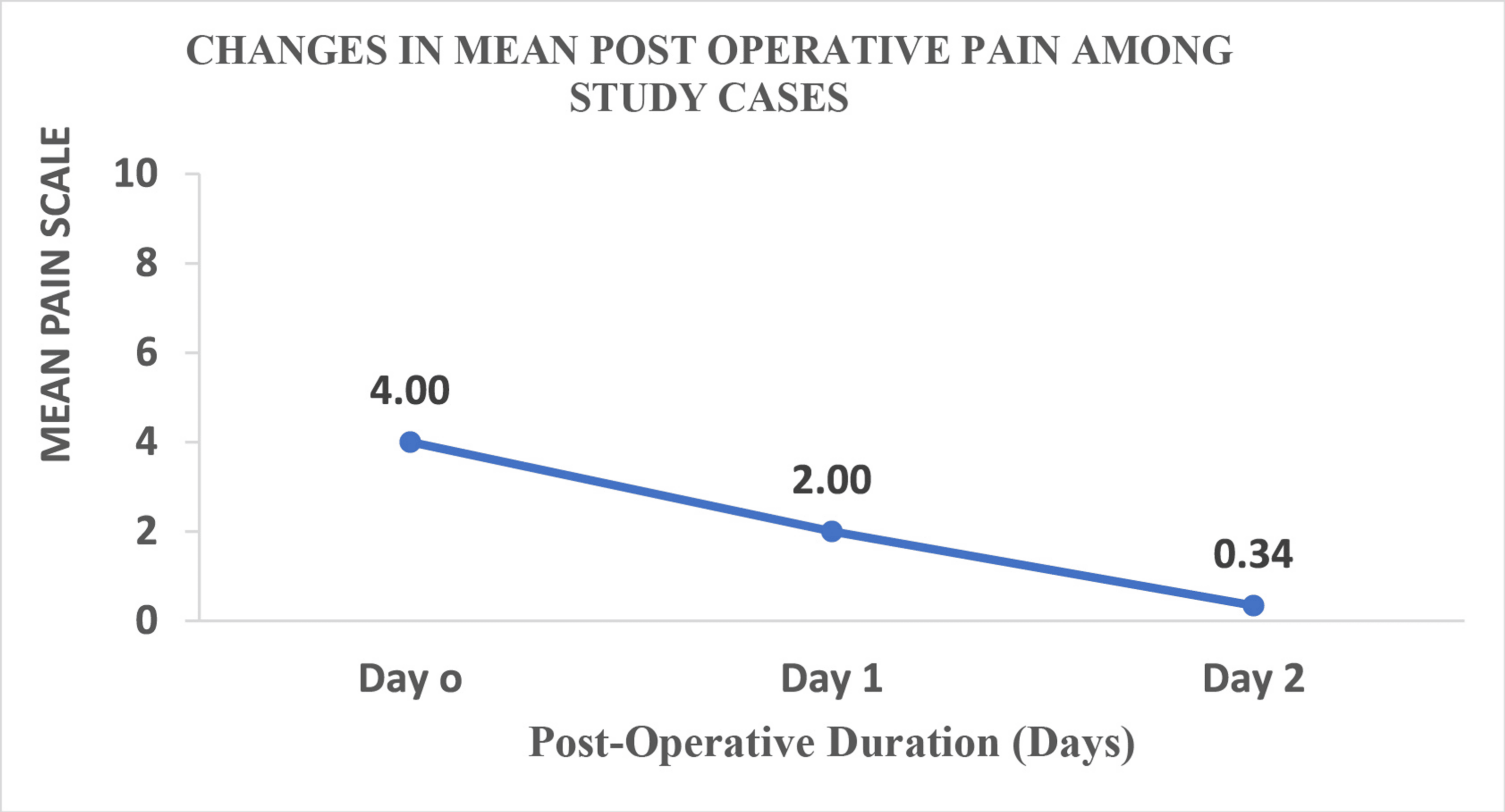
Changes in mean postoperative pain. By Wilcoxon sign rank test. Day 1: p = 0.001, i.e., <0.005. Day 2: p = 0.001, i.e., <0.005.

Patients were assessed by using the pain assessment scale as follows: 0 = no hurt; 2 = hurts a little bit; 4 = hurts little more; 6 = hurts even more; 8 = hurts whole lot; 10 = hurts worst. On pain assessment, it was noted that on day zero (D0), the average postoperative pain was 4, i.e., hurts a little more. On day one (D1), the mean pain score decreased significantly (p= 0.001, i.e., <0.005) to 50.0% from D0 and on day two (D2), the mean pain score showed a significant (p = 0.001, i.e., <0.005) fall of 91.5% from D0, as depicted in Figure 1.

Association between day of discharge and postoperative pain assessment

**Figure 2:**
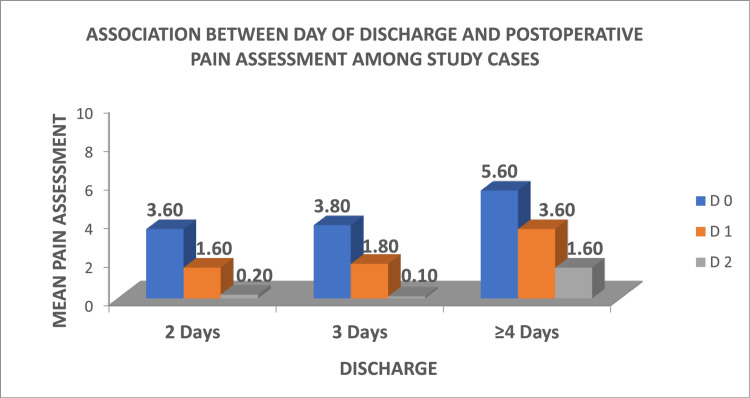
Association between day of discharge and postoperative pain assessment.

Ten patients with a mean pain assessment score of 3.60 at D0 were released on the second day, 20 patients with a mean score of 3.80 at D0 were released on the second day, and five patients with a mean score of 5.60 at D0 were released at ≥ four days, as illustrated in Figure 2.

Correlation between pain and day of discharge from the hospital

**Figure 3:**
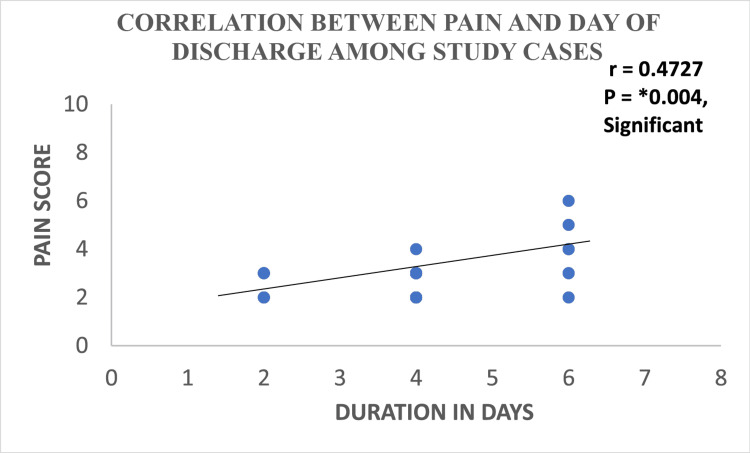
Correlation between pain and day of discharge from the hospital.

A significant (p = 0.004; r = 0.4727) correlation between pain at baseline and day of discharge was found. It was noted that patients with high mean postoperative pain scores (as per the pain assessment scale) at D0 had longer hospital stays as compared to the patients who had lower mean pain scores at D0, as demonstrated in Figure 3.

Association between operation time and complications

**Figure 4:**
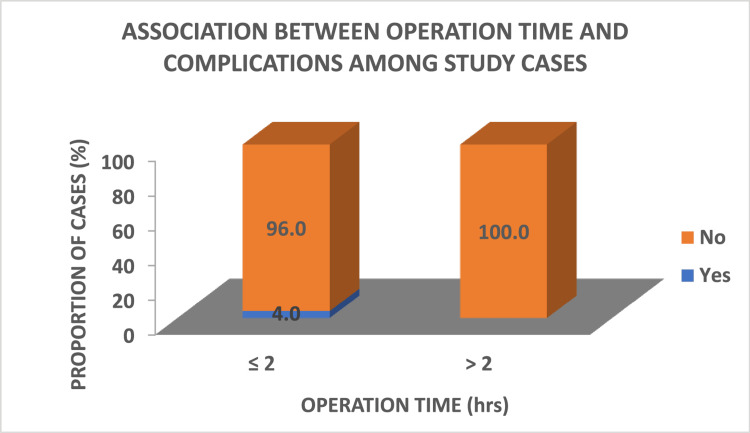
Association between operation time and complications. By Fisher's exact test. P = 1.00, not significant.

This study indicates that only one case (4%) with an operation time of ≤ two hours had complications and none of the patients with an operation time of > two hours developed any complications. However, a statistically significant difference (p = 1.00) was not observed, as depicted in Figure 4.

In the study, a total of two events (ureteral injury and vesicovaginal fistula) were reported on the third day of discharge, as shown in Table 4.

**Table 4:** Number of complications.

Complications	No. of events (N = 35)	Day of discharge, mean ± SD
Ureteral injury	01	3.00 ± 0.00
Vesicovaginal fistula	01	3.00 ± 0.00

It was noted that this patient who developed ureteral injury and vesicovaginal fistula was suffering from cervical fibroid measuring 4 * 3 cm. Because of this, there was less space in the pelvic zone, and hence ureter could not be lateralized making it prone to heat injury while performing the procedure.

## Discussion

In managing endometrial carcinoma, laparoscopic hysterectomy is the favoured option and it is an alternative to abdominal hysterectomy. Goel’s technique of 10 steps is designed to safely perform laparoscopic hysterectomy without the use of vaginal manipulators/myoma screws to avoid the risk of spread of malignancy through the systemic circulation. Goel’s technique is easily understood by surgeons and it has been well-received by the patients.

This single-centre retrospective study presented real-world evidence for using Goel’s technique for laparoscopy hysterectomy in 35 patients suffering from endometrial carcinoma. These patients were evaluated for the postoperative recovery journey three to four days after the operation.

In this study, 85.7% of patients were discharged on day two and day three, and 14.3% on day four or later. This study’s results reveal that the mean time to discharge the patients after surgery was 2.94 days. In the clinical study conducted by Soliman et al., 20 patients with endometrial carcinoma, particularly early-stage cancer, were treated with total laparoscopic hysterectomy (TLH) and pelvic lymphadenectomy additionally. Researchers reported the mean hospital stay of patients as 4.5 days [18]. Frigerioet al. reported 8.5 days and four days as mean hospital stays in the TAH and TLH groups, respectively [19]. In a study conducted by Zullo et al., 8.2 days and 4.1 days were reported as mean hospital stays in the TAH and TLH groups, respectively [20]. Our study observed a reduced hospital stay of 2.94 days with laparoscopic hysterectomy using Goel’s technique. This in turn comes about in diminishing the expenses of hospital stay and nursing care. This also signifies rapid patient recovery after adopting Goel’s technique while performing laparoscopic hysterectomy.

The mean operative time was 132.6 minutes in our study (range = 120-240 minutes). The mean operational time with TLH was 296.8 minutes in research by Soliman et al. [18]. Frigerio et al.'s study had a mean operative time of 175 minutes and 220 minutes in the TAH and TLH groups, respectively [19]. Zullo et al.'s study had a mean operative time of 135.3 minutes and 196.7 minutes in the TAH and TLH groups, respectively [20]. This is a changeable problem, as it heavily depends on the surgeon's expertise and learning curve. This holds true for the assistants as well, such as the nurses and technical support staff. Therefore, a skilled team approach to TLH expedites and secures the process.

The present study indicates that 2.9% of the cases had a ureteral injury and ureterovaginal fistula as a few complications and all these complications were resolved during the follow-up period. Not a single case had a blood transfusion and other complications, namely, bowel injury, bladder injury, and vesicovaginal fistula. One patient experienced delayed recovery as they developed postoperative ileus. The following instances of postoperative occurrences were documented in patients who had pelvic lymphadenectomy and TLH in research by Soliman et al.: 5% of the patients experienced postoperative bleeding, 5% had a pulmonary embolism, 20% had pyrexia development, and 5% had a chest infection [18]. As per the case series by Kohler et al., the following cases were reported in laparoscopically assisted radical hysterectomy and lymphadenectomy cases: 2.9% intraoperative (blood vessel or bowel injury) and 5.8% postoperative complications with an overall complication rate of 8.7% [21]. In the study by Frigerioet al., 10.9% of cases had the following complications in the TLH group: fever, pelvic abscess, urinary tract infection, and asymptomatic lymphocele. However, the same study reported that 29.9% of cases had the following complications in the TAH group: fascial disruptions, wound infections, myocardial infarction, ileus, asymptomatic lymphocele, and urinary tract infections [19].

Spilsbury et al. conducted a population-based retrospective observational analysis to assess the relative chances of complications during the admission for vaginal and abdominal hysterectomy [22]. Procedure-related haemorrhage (2.4%) was the most commonly recorded complication, followed by genitourinary disorders (1.9%), infection (1.6%), and urinary tract infections (1.6%). In another retrospective cohort study by Cici et al.,* *bowel injury occurred in 0.39% of cases in women undergoing hysterectomy. Bowel injury was found to be associated with older age, surgical indication of endometriosis, and abdominal surgical approach after adjusting for potential confounders [23]. In our study, very few complications were observed as compared to the review of the literature.

In the present study, after six months of follow-up, not a single patient developed any wound infection, dehiscence, or herniation. Soliman et al. reported one patient (5%) with wound infection in the laparoscopic hysterectomy group [18]. In an observational cohort study by Hur et al., 1.35% of patients reported dehiscence after a TLH [24].

In the current study, the mean postoperative pain decreased significantly by 50.0% and 91.5% on day one and day two, respectively, from day zero. In addition, a significant correlation between pain at baseline and the day of discharge was noted. It was found that patients with a high mean postoperative pain score (as per the pain assessment scale) on day zero had prolonged hospital stays as compared to the patients with a lower mean pain score on day zero. Overall, in this study, the use of Goel’s technique while performing laparoscopic hysterectomy resulted in reduced hospital stay and this parameter was represented as an indicator of rapid patient recovery as well. This further reduced the cost of nursing and in-patient care.

In the present study, we have used retrospective data of 35 patients suffering from endometrial cancer showing the benefits of using Goel’s technique while performing laparoscopic hysterectomy. However, we may be required to do a controlled randomized study in a larger population to confirm the benefits of using Goel’s technique.

## Conclusions

In managing endometrial carcinoma, laparoscopic-assisted vaginal hysterectomy is the favoured option. Goel’s technique carries out laparoscopic hysterectomy in a safe manner. Goel’s technique of laparoscopic-assisted vaginal hysterectomy is designed to safely perform laparoscopic hysterectomy without the use of vaginal manipulators/myoma screws to avoid the risk of spread of malignancy through the systemic circulation. In the present retrospective, observational, pilot study, the usage of Goel’s technique of laparoscopic hysterectomy helped early-stage endometrial cancer patients to recover faster and reduced hospital stays with fewer postoperative complications. This technique is contraindicated or caution is required in patients with cervical fibroid as it increases the probability of ureteral injury.
